# High-Pitch Computed Tomography Coronary Angiography—A New Dose-Saving Algorithm: Estimation of Radiation Exposure

**DOI:** 10.1155/2012/724129

**Published:** 2012-05-31

**Authors:** Dominik Ketelsen, Markus Buchgeister, Andreas Korn, Michael Fenchel, Bernhard Schmidt, Thomas G. Flohr, Christoph Thomas, Christoph Schabel, Ilias Tsiflikas, Roland Syha, Claus D. Claussen, Martin Heuschmid

**Affiliations:** ^1^Department of Diagnostic and Interventional Radiology, University Hospital of Tübingen, Hoppe-Seyler-Straße 3, 72076 Tübingen, Germany; ^2^Department of Mathematics, Physics & Chemistry, Beuth University of Applied Sciences, Luxemburger Straße 10, 13353 Berlin, Germany; ^3^Department of Diagnostic and Interventional Neuroradiology, University Hospital of Tübingen, Hoppe-Seyler-Straße 3, 72076 Tübingen, Germany; ^4^Division of Computed Tomography, Siemens Medical Solutions, An der Lände 1, 91301 Forchheim, Germany

## Abstract

*Purpose*. To estimate effective dose and organ equivalent doses of prospective ECG-triggered high-pitch CTCA. *Materials and Methods*. For dose measurements, an Alderson-Rando phantom equipped with thermoluminescent dosimeters was used. The effective dose was calculated according to ICRP 103. Exposure was performed on a second-generation dual-source scanner (SOMATOM Definition Flash, Siemens Medical Solutions, Germany). The following scan parameters were used: 320 mAs per rotation, 100 and 120 kV, pitch 3.4 for prospectively ECG-triggered high-pitch CTCA, scan range of 13.5 cm, collimation 64 × 2 × 0.6 mm with z-flying focal spot, gantry rotation time 280 ms, and simulated heart rate of 60 beats per minute. *Results*. Depending on the applied tube potential, the effective whole-body dose of the cardiac scan ranged from 1.1 mSv to 1.6 mSv and from 1.2 to 1.8 mSv for males and females, respectively. The radiosensitive breast tissue in the range of the primary beam caused an increased female-specific effective dose of 8.6%±0.3% compared to males. Decreasing the tube potential, a significant reduction of the effective dose of 35.8% and 36.0% can be achieved for males and females, respectively (*P* < 0.001). *Conclusion*. The radiologist and the CT technician should be aware of this new dose-saving strategy to keep the radiation exposure as low as reasonablly achievable.

## 1. Introduction

At present, computed tomography coronary angiography (CTCA) is an important, widely accepted diagnostic tool for the assessment of coronary artery disease. Several studies have shown the potential of different dose-saving strategies to keep the radiation exposure as low as reasonablly achievable. Hausleiter et al. reported in an international multicenter trial (PROTECTION I) a mean effective dose of 12 mSv in CTCA, ranging from 5 to 30 mSv [1]. Radiation exposure can be reduced substantially by currently available strategies, but these possibilities are used infrequently [1]. 

Since the introduction of a second-generation dual-source scanner system, a new scanning mode with increased table feed is available. Compared to retrospective ECG-gated and prospective ECG-triggered CTCA, this high-pitch, prospective triggered scanning mode has the potential for drastic dose reduction due to a gapless imaging of the heart within one heartbeat with no overlapping data acquisition.

The aim of the study was to estimate effective whole-body dose and organ equivalent doses of prospective ECG-triggered high-pitch CTCA.

## 2. Material and Methods

### 2.1. Dosimetry

The experiments were performed by using an anthropomorphic, hermaphrodite male phantom with breast phantom attachments (Alderson-Rando phantom; Alderson Research Laboratories Inc., Stanford, CT, USA). The phantom was equipped with 117 thermoluminescent dosimeters (TLDs) with dimension of 1 × 1 × 6 mm (TLD-100H, Bicon-Harshow, Radiation Measurement Products, Cleveland, OH, USA) to perform radiation exposure measurements [2–4].

By using a Philips Optimus 65 (Philips Medical Systems, PC Best, The Netherlands), a calibration of the 117 was calculated, which was defined by means of parallel exposure of 33 TLDs with a known radiation dose using 102 kV and 10 mA for 100 ms at a source-skin distance (SSD) of 100 cm. To minimize the Heel effect, wire markers in the field were avoided and all expositions were done in the same position with respect to the orientation of the X-ray tube. Crosschecked by an ionization dosimeter positioned in the same phantom depths, the reference TLDs were exposed with a dose of 1.081 mGy. No further correction factors were used as the calibration voltage is close to the CT tube voltage.

The evaluation of the irradiated TLDs was performed using a TLD reader (Model 5500 TLD Reader, Bicron Radiation Measurement Products, Solon, OH, USA) within 24 h after radiation exposure. The readout TLD values in nanocoulombs were multiplied by an individual calibration factor.

Dependent on the anatomical position of each organ 39 different positions in the Alderson-Rando Phantom were used to assess the organ doses. Due to measurement deviation, three TLDs were placed at each point of dose measurement to minimize bias. The number of TLDs allocated to different organ positions were as follows: 3 at the brain, thyroid gland, esophagus, thymus, heart, breast, stomach, upper colon, spleen, kidneys, adrenal glands, pancreas, small intestine, lower colon, urinary bladder, muscle tissue, red bone marrow, skin, ovaries, and testicles, 42 at the lung, and 15 at the liver.

The effective dose was calculated by summarizing the weighted organ doses according to the guidelines of ICRP 103 [5]. Radiation doses of simulated small organs (i.e., thyroid gland) were directly rated into the calculation. Doses of larger organs (i.e., lung) were determined by assessing the mean of measured TLDs from the entire organ.

To assess gender-specific differences, the testicles were used to measure the male-specific gonadal dose while radiation dose of the breast and the ovaries accounted to the female-specific radiation exposure. The breast phantom attachments could influence the calculation of the male-specific effective dose by increased soft tissue in the scan range. Nevertheless, evaluated values of effective dose using a hermaphrodite phantom are sufficiently precise for application in radiological radiation protection [5, 6].

### 2.2. Scan Protocols

Scans were performed on a second-generation dual-source CT scanner (SOMATOM Definition Flash, Siemens Medical Solutions, Forchheim, Germany). The following parameters were used: 320 mAs per rotation, 100 and 120 kV, pitch 3.4 for prospectively ECG-triggered CTCA (table feed 450 mm/s), scan range of 13.5 cm (four blocks of sequential detector coverage), collimation 64 × 2 × 0.6 mm with z-flying focal spot, and gantry rotation time 280 ms. The scanner software simulated a heart rate of 60 beats per minute. 60% of the RR-interval was set to trigger the scan. In high-pitch CTCA, the heart is scanned within one heartbeat. Data acquisition with two X-ray tubes and detectors allows a gapless imaging of the heart despite a high pitch of 3.4. Gaps in the data from the first measurement system resulting from the high pitch are filled completely with the data from the second measurement system that acquires the data a quarter rotation later [7].

### 2.3. Statistical Analysis

Normal distribution of measured organ-specific dose values of each scan protocol was assessed using the Kolmogorov-Smirnov test. Statistical significance between different scan protocols was evaluated using the two-tailed paired Student's *t*-test comparing the measured organ-specific dose values. A *P* value <0.05 was considered to be significant.

## 3. Results

Effective, gender-specific doses of scan protocols with 100 kV and 120 kV and reported CT dose index and dose length product are shown in Table 1.

Depending on the applied tube potential, the effective whole-body dose of the cardiac scan ranged from 1.1 mSv to 1.6 mSv and from 1.2 to 1.8 mSv for males and females, respectively.

Directly irradiated organs within the scan range received organ equivalent doses of up to 0.33 mSv to the lung and up to 0.13 mSv to the breast. The radiosensitive breast tissue in the range of the primary beam caused an increased female-specific effective dose of 8.6% ± 0.3% compared to males. The gonadal doses were <0.02 mSv in both scan protocols. Details about organ equivalent doses are displayed in Table 2.

A reduction of the tube potential from 120 kV to 100 kV significantly reduces the radiation exposure by 35.8% for males and 36.0% for females, respectively (*P* < 0.001).

## 4. Discussion

Several studies described the correlation between the pitch value and the radiation exposure in computed tomography coronary artery (CTCA). Typically, the pitch is less than 1, usually between 0.2 and 0.5 in first-generation dual-source CTCA resulting in relevant overlaps by advancing the table much less than one detector width during one scanner rotation [3, 7]. Thus, repeated exposure of the same heart region during several consecutive rotations causes an increase of radiation exposure.

The evaluated, prospective ECG-triggered high-pitch CTCA uses a pitch value of 3.4 resulting in a table feed of 450 mm/s with no overlapping data acquisition by filling the gaps in the data of the spiral acquisition of the first measurement system with the data from the second measurement system one-quarter rotation later. The whole heart is completely scanned within one heart cycle, that is, the topmost image will be acquired at the set trigger point and subsequent images display the heart in later phases of the heart cycle [8–11].

In this study, we determined the effective radiation exposure of prospective ECG-triggered high-pitch CTCA depending on the gender and the used tube voltage. The results are concordant to recently reported dose values from Goetti et al. [8]. The radiation exposure is substantially lower than other reported dose values of retrospective ECG-gated or prospective ECG-triggered CTCA. A comparative study of different dose-saving techniques reports effective dose values of 5.8 mSv to 16.0 mSv for retrospective ECG-gated protocols without ECG-pulsing, and 4.2 mSv to 9.8 mSv for protocols with different ECG-pulsing techniques and 2.8 mSv to 4.3 mSv for prospective ECG-triggered, the so-called step-and-shoot, CTCA [12].

A further radiation protective effect comprises the acquisition of less overlapping data. This results in reduction of the organ equivalent dose of the extremely radiosensitive female breast tissue, which is always in the scan range, but rarely organ of interest. In high-pitch CTCA, the female-specific effective dose is higher compared to male effective dose measurements by an average of up to 8.6%. In conventional overlapping CTCA data acquisition, however, females received increased effective dose values of up to 70% [6, 12–14].

By decreasing the tube voltage, the effective dose can be further reduced by up to 36.0% in high-pitch CTCA. However, changes in tube voltage are complex and affect image noise as well as tissue contrast. Nevertheless, several studies confirmed a sufficient image quality of 100 kV CTCA protocols in slender patients with a body mass index lower than 30 kg/m^2^ [15].

A possible limitation of the study is the lack to determine image quality as an additional parameter of the technique. However, initial feasibility studies reported an excellent image quality in a selected patient population [9, 15]. High-pitch CTCA shows equivalent image quality in patients with low and stable heart rates up to 60 bpm compared to prospective ECG-triggered and retrospective ECG-gated CTCA. A diagnostic image quality can be achieved in 97% of coronary segments with high-pitch CTCA [16–18]. These studies recommend this prospective, R-wave triggered, high-pitch scanning mode for patients with low and regular heart rates (<55–60 beats per minute) to acquire the complete dataset before the onset of atrial contraction [7, 9, 10].

## 5. Conclusion

In conclusion, the prospective ECG-triggered high-pitch CTCA has the potential to scan the heart with an effective dose between 1 mSv and 2 mSv, which is substantially lower than previously described CTCA scanning modes. The radiologist and the CT technician should be aware of this new dose-saving strategy to keep the radiation exposure as low as reasonablly achievable.

## Figures and Tables

**Table 1:** Effective radiation exposure of high-pitch CTCA using 100 kV and 120 kV.

Protocol	Effective dose male [mSv]	Effective dose female [mSv]	Total mAs	CTDI_Vol_ (mGy)	DLP (mGy cm)
1 (100 kV)	1.1	1.2	688	3.1	56
2 (120 kV)	1.6	1.8	689	5.2	95

CTDI_Vol_: volume CT dose index; DLP: dose-length product.

**Table 2:** Measured organ equivalent doses of high-pitch CTCA using different tube voltages (mSv).

Organ/tissue	100 kV	120 kV
Thyroid gland	0.03	0.04
Esophagus	0.05	0.05
Lung	0.20	0.33
Breast	0.07	0.13
Stomach	0.45	0.67
Liver	0.06	0.12
Colon	0.03	0.03
Urinary bladder	0.00	0.00
Red bone marrow	0.01	0.02
Skeleton	0.00	0.00
Skin	0.00	0.01
Male gonads	0.00	0.00
Female gonads	0.01	0.01
Remaining organs	0.22	0.37
